# Fluticasone Propionate Protects against Ozone-Induced Airway Inflammation and Modified Immune Cell Activation Markers in Healthy Volunteers

**DOI:** 10.1289/ehp.10981

**Published:** 2008-02-28

**Authors:** Neil E. Alexis, John C. Lay, Angela Haczku, Henry Gong, William Linn, Milan J. Hazucha, Brad Harris, Ruth Tal-Singer, David B. Peden

**Affiliations:** 1 Center for Environmental Medicine, Asthma and Lung Biology, and; 2 Department of Medicine, University of North Carolina, Chapel Hill, North Carolina, USA; 3 Department of Medicine, University of Pennsylvania, Philadelphia, Pennsylvania, USA; 4 Department of Medicine, University of California, Los Angeles, California, USA; 5 Environmental Health Service, Rancho Los Amigos National Rehabilitation Center, Downey, California, USA; 6 GlaxoSmithKline, King of Prussia, Pennsylvania, USA

**Keywords:** inhaled corticosteroids, innate immune markers, ozone, sputum neutrophils

## Abstract

**Background:**

Ozone exposure induces airway neutrophilia and modifies innate immune monocytic cell-surface phenotypes in healthy individuals. High-dose inhaled corticosteroids can reduce O_3_-induced airway inflammation, but their effect on innate immune activation is unknown.

**Objectives:**

We used a human O_3_ inhalation challenge model to examine the effectiveness of clinically relevant doses of inhaled corticosteroids on airway inflammation and markers of innate immune activation in healthy volunteers.

**Methods:**

Seventeen O_3_-responsive subjects [> 10% increase in the percentage of polymorphonuclear leukocytes (PMNs) in sputum, PMNs per milligram vs. baseline sputum] received placebo, or either a single therapeutic dose (0.5 mg) or a high dose (2 mg) of inhaled fluticasone proprionate (FP) 1 hr before a 3-hr O_3_ challenge (0.25 ppm) on three separate occasions at least 2 weeks apart. Lung function, exhaled nitric oxide, sputum, and systemic biomarkers were assessed 1–5 hr after the O_3_ challenge. To determine the effect of FP on cellular function, we assessed sputum cells from seven subjects by flow cytometry for cell-surface marker activation.

**Results:**

FP had no effect on O_3_-induced lung function decline. Compared with placebo, 0.5 mg and 2 mg FP reduced O_3_-induced sputum neutrophilia by 18% and 35%, respectively. A similar effect was observed on the airway-specific serum biomarker Clara cell protein 16 (CCP16). Furthermore, FP pretreatment significantly reduced O_3_-induced modification of CD11b, mCD14, CD64, CD16, HLA-DR, and CD86 on sputum monocytes in a dose-dependent manner.

**Conclusions:**

This study confirmed and extended data demonstrating the protective effect of FP against O_3_-induced airway inflammation and immune cell activation.

Ozone is a commonly encountered environmental air pollutant. In epidemiologic investigations, exposure to increased levels of ambient air O_3_ has been associated with exacerbations of asthma, chronic obstructive pulmonary disease (COPD), and pneumonia, generally 24–48 hr after exposure occurs (Bernstein et al. 2004; Peden 2001). Controlled chamber exposures to O_3_ cause an influx of neutrophils to the airway and a decrease in lung function, although these two effects do not correlate with each other, indicating that separate mechanisms account for these effects (Bernstein et al. 2004). O_3_ exposure also causes increased responsiveness to allergen in allergic asthmatics (Peden 2001). We have recently observed that O_3_ exposure can also result in increased expression of CD11b, CD14, CD16, CD80, CD86, and HLA-DR on airway dendritic cells (DCs), monocytes, and macrophages (Alexis et al. 2004b). It has been suggested that the action of O_3_ on airway neutrophils, monocytes, and macrophages accounts for much of the disease outcomes associated with O_3_ exposure. These inflammatory events also mimic the type of inflammation that occurs with acute viral and bacterial infection and exacerbations of asthma and COPD (Maneechotesuwan et al. 2007; Pauwels 2004).

Together, these observations suggest that O_3_ challenge may be a useful controlled human disease model for screening novel anti-inflammatory pharmaceutical agents in phase I proof-of-concept trials. Holz et al. (2005) tested the utility of a 0.25-ppm O_3_ challenge as a drug efficacy screen, using a single pre-treatment dose of the established anti-inflammatory agents fluticasone propionate (FP) and oral prednisolone as test agents in a randomized three-arm crossover study in 18 healthy subjects comparing the effect of these two treatments with that of placebo on O_3_-induced airway inflammation. Holz et al. (2005) reported that, compared with placebo, pretreatment with 2 mg inhaled FP and 50 mg oral prednisolone resulted in a significant reduction in post-O_3_ sputum neutrophils per milliliter (by 62% and 64%, respectively) and myeloperoxidase (MPO; by 55% and 42%, respectively). These results demonstrated that corticosteroids do inhibit the proinflammatory actions of O_3_.

In the present study, we sought to extend these observations by comparing the effect of a single administration of a high dose of inhaled FP (2 mg) with a dose that is employed in clinical practice for asthma and COPD (0.5 mg) and placebo. Given the importance that monocytes, macrophages, and DCs likely have in the pathophysiology of O_3_-induced exacerbations of disease, we also examined the effect of these treatments on expression of CD11b/CR3, mCD14, CD16/FcγRIII, CD64/FcγRI, CD86/B7, and HLA-DR on monocytes, macrophages, and DCs recovered from airway sputum. Clara cell protein 16 (CCP16) and surfactant protein D (SP-D) are innate immune molecules and products of airway epithelial cells (Haczku 2006) that can be released to the circulation during lung injury (Holz et al. 2005). CCP16 is induced in the serum of subjects exposed to O_3_ challenge (Blomberg et al. 2003). We have previously shown that SP-D levels in the lung are significantly altered after O_3_ inhalation in mice (Kierstein et al. 2006), but whether similar changes can be detected in the human serum is not known. Thus, we evaluated CCP16 and SP-D for their potential utility as serum biomarkers for assessing the effects of inhaled corticosteroids on O_3_ injury in the respiratory tract.

## Materials and Methods

### Subjects

Seventeen (nine male and eight female) nonsmoking healthy volunteers (10 from the Center for Environmental Medicine, Asthma and Lung Biology; 7 from Rancho Los Amigos National Rehabilitation Center) between 18 and 50 years of age (age, 26.4 ± 7.4 years, mean ± SD; body mass index, 20–30 kg/m^2^) were recruited for this study. All subjects underwent a thorough physical examination and had no history of cardiovascular or chronic respiratory disease and were free of upper or lower respiratory tract infection at least 4 weeks before study participation. All subjects had a forced expiratory volume in 1 sec (FEV_1_) of at least 80% predicted for a normal population of similar weight and height. A positive urine pregnancy test resulted in exclusion of female subjects from the study. The use of prescription drugs, over-the-counter medication (e.g., aspirin and nonsteroidal anti-inflammatory drugs), vitamins, antioxidants, and dietary supplements was not permitted for the duration of the study. All study participants were able to produce an adequate sputum sample (≥ 1 × 10^6^ total cells, ≥ 50% cell viability, ≤ 20% squamous epithelial cells) as measured on their first baseline visit (sputum with no O_3_ exposure), and all were responsive to O_3_ (defined as ≥ 10% increase in total and percent sputum neutrophils) (Holz et al. 2005) after exposure to 0.25 ppm O_3_ for 3 hr with intermittent moderate exercise (ventilation_expiratory_ = 12.5 L/min/m^2^ body surface area) as measured on the second study visit. The study was approved by the Committee on the Protection of the Rights of Human Subjects, School of Medicine, University of North Carolina at Chapel Hill, and by the Institutional Review Board at the Rancho Los Amigos National Rehabilitation Center. Informed written consent was obtained from all subjects before their participation in the study.

### Study design

**Figure 1:**
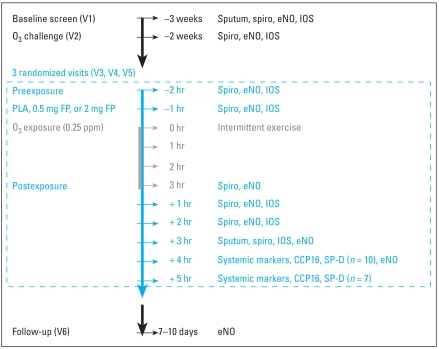
Schematic of the study design. Abbreviations: PLA, placebo; Spiro, spirometry; V, visit. The study was a double-blinded, randomized, cross-over design with a 2-week washout period between visits. Except for the first visit (screen) and last visit (follow-up), all visits included an O_3_ exposure (0.25 ppm, 3 hr).

This was a double-blind, placebo-controlled, single-dose, randomized, three-period crossover study conducted at two sites. Controlled O_3_ exposures were performed in comparable chamber setups at both the University of North Carolina, Chapel Hill and the Rancho Los Amigos facility (Alexis et al. 2004a; Gong et al. 1998). All subjects underwent 3-hr exposures to 0.25 ppm O_3_ with intermittent moderate exercise (15 min rest, 15 min exercise at 12.5 L/min/m^2^ body surface area) at screening visit 2 and each study session thereafter (visits 3–5). Based on FP half-life and washout of sputum neutrophils after O_3_ exposure (Holz et al. 2005), O_3_ exposures were separated by a minimum of 2 weeks to avoid carryover effects. FEV_1_ and forced vital capacity (FVC) were also measured for the purpose of assessing subject safety. Sputum induction was performed at screening visits and at 3 hr after the conclusion of each O_3_ exposure (i.e., post-exposure). Sputum was analyzed for total and differential leukocyte count and fluid-phase components and in a subset of subjects (*n* = 7) for cell-surface phenotypes and cell function by flow cytometry. The study design, including measurement time points, is depicted in Figure 1. FP was provided as a metered dry powder inhaler (Diskus; GlaxoSmithKline, Research Triangle Park, NC). Each Diskus device contained 60 × 0.5 mg doses of FP. A matching placebo Diskus was also provided. Subjects were randomized to receive one of the following treatment regimens: *a*) 0.5 mg FP (one inhalation of 0.5 mg FP plus three inhalations of placebo); *b*) 2 mg FP (one inhalation of 0.5 mg FP plus three inhalations of 0.5 mg FP); and *c*) placebo (one inhalation of placebo plus three inhalations of placebo). The study staff observed each subject using the Diskus during clinic visits to ensure that the device was used correctly.

### Pulmonary function

We used both spirometry and impulse oscillometry (IOS) to assess lung function status in subjects. Spirometry was assessed at preexposure, immediately postexposure, and then at 1-hr intervals for 3 hr. IOS was assessed at pre-exposure, and then hourly for 3 hr beginning 1 hr postexposure. Airway resistance and airway reactance were determined by IOS (Jaeger MS-IOS and LAB Manager Software, version 4.53.2; Jaeger, Hoechberg, Germany) using the recommended techniques of the manufacturer and as previously described (Singh et al. 2006). Real-time recordings of mouth pressure and flow signals pulsed through 5- to 35-Hz spectrum were superimposed on tracings of tidal breathing and displayed on a computer screen. Measurements of total respiratory resistance, resonant frequency (*F*_res_), reactance at 5 Hz, and low-frequency reactance area (area of reactance integrated from 5 Hz up to *F*_res_) were recorded at the 5−, 10−, 15−, and 20-min time points after the IOS test challenge. Spirometry was performed according to current American Thoracic Society spirometry standards (Enright 2003).

### Sputum induction and processing and fluid-phase analyses

Subjects provided an induced sputum sample during the screening visit and at 3 hr post-O_3_ exposure. The sputum induction and processing methods have been previously described in detail (Alexis et al. 2003, 2006). In brief, three 7-min inhalation periods of nebulized hypertonic saline (3%, 4%, 5%; Devilbiss UltraNeb 99 ultrasonic nebulizer; Sunrise Medical, Somerset, Somerset, PA) were followed by expectoration of sputum into a sterile specimen cup. Sputum cell aggregates (cellular mucus plugs) were macroscopically identified and manually selected from their surrounding fluid and treated with 0.1% dithiothreitol (DTT; Sputolysin, Calbiochem, San Diego, CA). Total cell counts and cell viability were determined using a Neubauer hemacytometer and trypan blue (Sigma Chemical Co., St. Louis, MO) exclusion staining. Differential cell counts were analyzed using the Hema-Stain-3 kit (Fisher Diagnostics, Middletown, VA). Aliquots of DTT-treated sputum supernatant were immediately frozen and stored at −80°C for later analysis of MPO and total protein by multiplex assay (Pierce Biotechnology, Rockford, IL). All soluble factors (cytokines and chemokines) in sputum (MPO, total protein) were analyzed by a contract laboratory (HFL, Fordham, UK) using validated commercial enzyme-linked immunosorbent assay (ELISA) kits. All compounds were validated in the presence of DTT. The limits of detection after dilution (to minimize potential effects of DTT and to achieve sufficient volume for measurements) were 40 μg/mL for total protein (Dojindo Molecular Technologies, Inc., Gaithersburg, MD) and 36 ng/mL for MPO (Immundiagnostik, Bensheim, Germany).

For a subset of samples, remaining cells were resuspended in Hank’s balanced salt solution and kept on ice for immediate use in flow cytometric assays for selected cell-surface molecules and phagocytosis.

### Systemic biomarkers

Venipuncture was performed at 4 or 5 hr after O_3_ exposures to obtain serum for Multiplex systemic biomarker analysis of tumor necrosis factor-α (TNF-α), interferon-γ (INF-γ), interleukin-6 (IL-6), IL-1β, IL-1Ra, IL-17, eotaxin, and IL-12P40 using fluorometric custom-designed validated Multiplex kits (Pathway Diagnostics, Malibu, CA). CCP16 and SP-D were assayed using commercially available ELISA kits (Biovendor, Candler, NC) according to the manufacturer’s instructions.

### Flow cytometry and immunofluorescent staining

All flow cytometry acquisitions and analyses (surface markers, phagocytosis) were performed as previously described (Alexis et al. 2000a) using a FACSort flow cyto-meter (Becton Dickinson, Franklin Lakes, NJ) and CellQuest Pro v5.3 software (Becton Dickinson).

### Cell-surface phenotypes

Immuno-fluorescent staining and flow-cytometry methodology have been described in detail in previous publications (Alexis et al. 2003, 2006). In brief, cells (100 μL, 1 × 10^6^/mL) were incubated with 10 μL fluorochrome-labeled monoclonal antibodies, washed in Dulbecco’s phosphate-buffered saline (DPBS), fixed with 0.5% paraformaldehyde in DPBS, and analyzed by flow cytometry within 48 hr of fixation. Viable macrophages, monocytes, neutrophils, lymphocytes, and DCs in sputum were initially identified and gated on the basis of light-scatter properties and positive expression for CD45 (pan-leukocyte marker). Cell populations were then confirmed by positive staining with CD16 (neutrophils), mCD14 (monocytes), HLA-DR (macrophages), HLA-DR/CD86 (DCs), and CD3 (lymphocytes). The acquired data were analyzed using CellQuest Pro v5.3 software, and results were expressed as a rightward shift from control in mean fluorescence intensity (MFI) on histogram analysis. Control cells were incubated with appropriately labeled isotypic control antibodies. Surface markers analyzed included markers of innate (CD11b/CR3, mCD14/LPS receptor, CD16/FcγRIII, CD64/FcγRI) and adaptive (HLA-DR/MHC class II, and CD86/B7.2 co-receptor) immune function. All monoclonal antibodies were purchased from Beckman Coulter Corporation (Miami, FL).

### Phagocytosis

We analyzed phagocytosis using fluorescein isothiocyanate–labeled IgG-opsonized *Saccharomyces cerevisiae* zymosan-A BioParticles (Molecular Probes, Eugene, OR) as previously described (Alexis et al. 2003, 2006). All samples were analyzed by flow cytometry within 24–48 hr of fixation in 1% paraformaldehyde. Particle uptake was displayed on histograms and identified as a rightward shift in MFI of the phagocytic population versus autofluorescence of the unlabeled control cells.

### Exhaled nitric oxide

We measured exhaled NO (eNO) levels preexposure, immediately after exposure, and then at 1-hr intervals for 4 hr according to standardized procedures jointly recommended by the American Thoracic Society and the European Respiratory Society (2005) using a NIOX NO analyzer (Aerocrine AB, Solna, Sweden).

### Statistical analysis

To determine the total number of neutrophils and fluid-phase markers (MPO, protein) in induced sputum 6 hr postchallenge, we analyzed data following a natural logarithmic transformation using a mixed effects model, with period and treatment fitted as fixed effects and subject as a random effect. The suitability of the transformation was assessed by examining the model residuals. Treatment effects were evaluated in terms of treatment ratios and were calculated as the antilog for the differences between the least squares means; 95% confidence intervals (CIs) were determined using pooled estimates of variance for the least squares means difference and then antilogged.

For assessment of differences between specific treatment conditions (postscreen O_3_ challenge vs. placebo vs. both doses of FP) for CCP16, SP-D, systemic cytokines, and cell-surface marker expression in the subset (*n* = 7) of volunteers studied at University of North Carolina, Chapel Hill, we used nonparametric one-way analysis of variance for repeated measures (Friedman test) and Dunn’s post hoc analysis of specific pairs of variables. An overall significance level of *p* < 0.05 was considered to be significant. All values are expressed as mean ± SE. We used GraphPad Prism 3.1 software (GraphPad Software, Inc., San Diego, CA) for statistical analysis.

## Results

### Patient demographics and overall safety

Seventeen volunteers participated in the study; patient demographics are outlined in Table 1. No serious adverse events were reported during this study.

### Effects of FP on 0.25 ppm O_3_-induced changes in pulmonary function

O_3_ exposure caused decreases in FVC and FEV_1_ during all exposures. Decrements in FVC and FEV_1_ were evident immediately after O_3_ exposure during placebo, 0.5 mg, and 2 mg FP treatments but were subsiding by 1 hr post-exposure for each treatment condition (Table 2). Decrements in FVC and FEV_1_ were minimal by 3 hr postexposure (Table 2). Neither dose of FP had a statistically significant effect on O_3_-induced lung function changes compared with placebo. No consistent O_3_-induced changes were observed in IOS end points at any postexposure time point (Table 2).

### Effects of FP on 0.25 ppm O_3_-induced changes in sputum neutrophils and fluid-phase markers of neutrophil activation (MPO, total protein)

**Figure 2:**
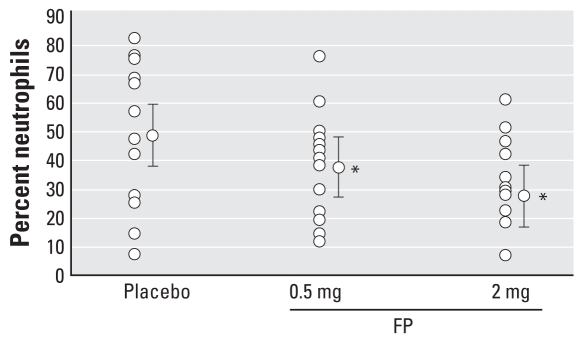
The percent sputum neutrophils after O_3_ exposure for each pretreatment dose of FP (0.5 or 2 mg) or placebo. *p < 0.05 compared with placebo.

Analysis of percent neutrophil levels post-O_3_ challenge yielded evidence of a statistically significant difference for both active treatments (0.5 mg and 2 mg FP) relative to placebo. Mean ± SE levels of percent polymorphonuclear leukocytes (PMNs) for placebo and 0.5 mg and 2 mg FP were 54 ± 5.4%, 44 ± 4.5%, and 35 ± 3.6%, respectively (Figure 2), which reflect an 18% and 35% reduction in sputum percent PMNs for 0.5 mg and 2 mg FP, respectively. The data indicate a dose–response pattern.

FP also affected the relatively more variable total number of neutrophils/mL. The mean (95% CI) numbers of PMNs/mL were 66.05 × 10^4^ cells/mL (34.78–125.41 cells/mL), 56.87 × 10^4^ cells/mL (30.15–107.27 cells/mL), and 37.49 × 10^4^ cells/mL (19.89–70.68 cells/mL) for placebo, 0.5 mg FP, and 2 mg FP, respectively, 3 hr post-O_3_ exposure. These values reflected 14% fewer neutrophils in sputum when subjects were pretreated with 0.5 mg FP and statistically significantly (*p* < 0.05) fewer neutrophils (43%) when pretreated with 2 mg FP, indicating a dose–response effect on the total number of neutrophils per milliliter. In terms of variability, the neutrophil responses on the O_3_/placebo visit versus the O_3_-only visit were very similar for both percent neutrophils (mean ± SE, 54 ± 5% vs. 55 ± 5%) and the absolute number of neutrophils per milligram sputum [mean (95% CI), 66.05 × 10^4^ cells/mL (34.78 to 125.41 cells/mL) vs. 62.20 × 10^4^ cells/mL (−10.17 to 312.97 cells/mL), respectively]. Other than percent macrophages, FP exerted no statistically significant effect on total leukocytes per milliliter or total and percent eosinophils, lymphocytes, and bronchial epithelial cells. Relative to placebo, we observed a 24% and 48% increase in percent macrophages with 0.5 mg and 2 mg FP, respectively.

We observed no statistically significant treatment effect of 0.5 mg or 2 mg FP on MPO or total protein levels in sputum. There was, however, borderline evidence of a difference in levels of the MPO/total protein ratio relative to placebo for 2 mg FP. We observed, on average, reductions of 18% and 43% in the MPO/total protein ratio for 0.5 mg and 2 mg FP, respectively, suggesting a dose–response relationship.

### Effects of FP on 0.25 ppm O_3_-induced changes in surface marker expression and phagocytosis on sputum monocytes, macrophages, DCs, and neutrophils

**Figure 3:**
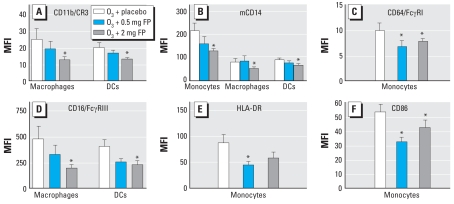
Expression (MFI; mean ± SE) of cell-surface phenotypes on sputum monocytic cells and DCs after O_3_ exposure with 0.5 mg FP, 2 mg FP, or placebo pretreatment. (*A*) CD11b/CR3. (*B*) mCD14. (*C*) CD64/FcγRI. (*D*) CD16/FcγRIII. (*E*) HLA-DR. (*F*) CD86. Only results in which at least one dose of FP resulted in a change in surface marker expression compared with placebo are shown. *p < 0.05 for CD11b, mCD14, CD64, CD16, and CD86 compared with 2 mg FP and for HLA-DR and CD86 with compared with 0.5 mg FP.

Figure 3 shows the effect of 0.5 and 2 mg pretreatments with FP on O_3_-induced changes in the cell-surface markers CD11b, mCD14, CD64, CD16, HLA-DR, and CD86 on monocytes, macrophages, and DCs. Baseline (i.e., no O_3_ exposure) sputum cell-surface marker values (MFI; mean ± SE) from a different cohort of healthy volunteers (*n* = 15) were as follows: for CD11b, 21 ± 8 macrophages, 16 ± 3 DCs; for mCD14, 65 ± 16 macrophages, 59 ± 14 monocytes, 61 ± 11 DCs; for CD64, 5 ± 1 monocytes; for CD16, 238 ± 44, macrophages, 195 ± 28 DCs; for HLA-DR: 31 ± 5 monocytes; and for CD86, 22 ± 4 monocytes (Lay et al. 2007). Compared with the O_3_-only condition in this study (data not shown), baseline expression of these surface markers was significantly (*p* < 0.05) lower, indicating that O_3_ causes an up-regulation of these cell-surface phenotypes.

In general, 2 mg FP exerted a statistically significant effect on post-O_3_ surface marker expression relative to placebo treatment. There was also a similar trend after the 0.5 mg dose, which suggests a dose–response effect of FP on O_3_-induced changes in monocytic cell-surface markers. We also observed a significant decrease in CD16/FcγRIII expression on neutrophils after 2.0 mg FP compared with placebo (MFI, 406 ± 64 vs. 515 ± 72; *p* < 0.05). We observed no significant drug effect of 0.5 mg or 2 mg FP versus placebo on sputum cells as measured by MFI (mean ± SE): for phagocytosis for macrophages, 478 ± 76 and 606 ± 102 versus 400 ± 50; for monocytes, 348 ± 46 and 365 ± 55 versus 292 ± 39; and for neutrophils, 296 ± 48 and 418 ± 87 versus 270 ± 29.

### Effects of FP on 0.25 ppm O_3_-induced changes in serum CCP16, SP-D, eNO, and other systemic biomarkers

**Figure 4:**
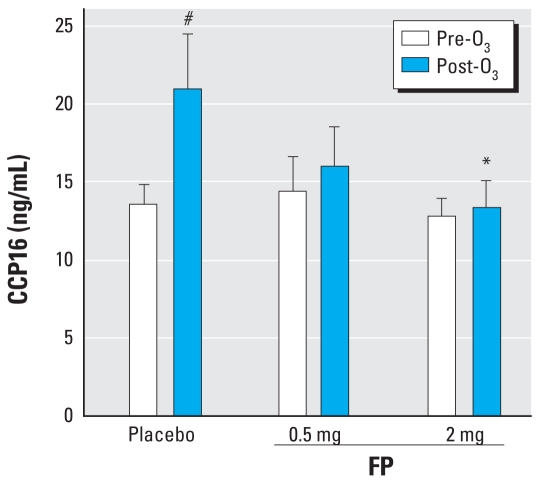
CCP16 levels (mean ± SE) in serum pre-O_3_ and 8 hr post-O_3_ for placebo and 0.5 and 2 mg FP *p < 0.05 compared with placebo. #*p* < 0.05 for post-O_3_ compared with pre-O_3_.

To determine whether serum levels of the airway epithelial cell products SP-D and CCP16 would reflect inflammatory airway changes after O_3_ exposure, we measured the concentration of these molecules at baseline and after each O_3_ inhalation session in a subset of seven subjects 5 hr after O_3_ exposure. Our results showed that serum CCP16 levels were statistically significantly increased after O_3_ inhalation and that pretreatment with 2 mg FP statistically significantly inhibited this effect compared with placebo (Figure 4). The effects of FP on CCP16 were dose dependent. SP-D levels were not statistically significantly altered pre-versus post-O_3_ exposure (mean ± SE, 61 ± 6 ng/mL vs. 55 ± 5.4 ng/mL) and were not significantly affected by 0.5 mg FP (53 ± 5 ng/mL) or 2 mg FP (64 ± 5 ng/mL) compared with placebo (55 ± 5 ng/mL).

Statistical analysis of other systemic bio-markers or eNO yielded no clear changes induced by O_3_ exposure. No significant effects on systemic cytokines (IL-6, IL-12P40, IL-15, IL-17, IL-1β, IL-1Ra, INF-γ, TNF-α), mediators (MPO, eotaxin), or eNO (Table 2) were observed after 0.5 mg or 2 mg FP versus placebo. For eNO, levels at 1, 2, and 3 hr (Table 2) postexposure were not statistically significantly different from one another.

## Discussion

Numerous laboratory studies of healthy young individuals exposed to O_3_ at a dose comparable to that used in the present study have demonstrated decrements in spirometric lung function (Holz et al. 1999, 2005; McDonnell et al. 1997; Nightingale et al. 2000). A study similar to this one in terms of the cohort characteristics, O_3_ concentration, and ventilation rate also reported similar postexposure decrements in FVC and FEV_1_ (Holz et al. 1999). In the present study we found that pre-treatment with therapeutic doses of FP had no significant protective effect on spirometric response, which is in agreement with the finding of Nightingale et al. (2000). FP did, however, inhibit inflammatory cell (neutrophils, PMNs) influx to the airways induced by a 3-hr exposure to 0.25 ppm O_3_ in a dose-dependent manner. The lack of correlation between spirometry and airway inflammation after O_3_ has been well documented (Blomberg et al. 1999; Hazucha et al. 1996), so our finding with FP in this regard was not surprising.

We observed a significant inhibition of the percent PMNs present in airway sputum after O_3_ challenge with either 0.5 mg or 2 mg FP pretreatment, and a significant reduction and a trend for reduction in the number of sputum neutrophils per milliliter post-O_3_ with 2 mg and 0.5 mg FP, respectively. Furthermore, we showed that serum CCP16 is a valuable systemic marker of the inflammatory state of the lung and is responsive to the effects of inhaled FP. We also observed evidence of diminished neutrophil activation with 2 mg FP, as it decreased the expression of CD16, a marker of neutrophil activation, compared with placebo. This observation coincided with a reduced MPO/total protein ratio with 2 mg FP, supporting the notion of reduced neutrophil activation. Taken together with previously published results (Holz et al. 2005), our results indicate that O_3_ challenge in healthy individuals is a useful model for screening novel anti-inflammatory agents designed for treatment of airway diseases that have elevated neutrophils as a principal component of their airway inflammation. These include a subtype of severe asthma with minimal airway eosinophils (Louis et al. 2000; Wenzel 2003; Wenzel et al. 1999), as well as COPD during an exacerbation (Hill et al. 1999; Stockley 1998).

An important feature of our study design was that we limited volunteer recruitment to persons with documented responsiveness to O_3_, defined as a minimum of a 10% increase in air-way PMNs after a screening O_3_ challenge, to enable the assessment of FP. Nightingale et al. (2000) failed to observe an effect when they examined the effect of 2 weeks of treatment with 800 μg inhaled budesonide twice daily on O_3_-induced neutrophilia in normal volunteers. In our study, although there was a significant effect of O_3_ alone on percent PMNs, a substantial number of persons examined failed to have an absolute neutrophil response (using neutrophils per milligram sputum as a measure) after placebo treatment. Thus, it is possible that Nightingale et al.’s (2000) results were influenced by a study population that included a high proportion of O_3_ “non-responders.” In contrast, Vagaggini et al. (2001) examined the effect of 4 weeks of pre-treatment with 400 μg inhaled budesonide twice daily on O_3_-induced neutrophilia in asthmatics, and reported a significant decrease in airway neutrophils present 6 hr after 0.27 ppm O_3_ challenge compared with placebo pretreatment; most volunteers in the Vagaggini et al. (2001) study appeared to be O_3_ responsive. Furthermore, the objective of the present study was not to test the efficacy of the O_3_ model, but rather to determine whether clinically relevant doses of FP could be assessed to support subsequent larger studies in subjects with preexisting airway disease.

In addition to its effects on airway neutrophilia, we have recently reported that O_3_ challenge (0.4 ppm, 2 hr) causes an increase in expression of cell-surface phenotypes CD11b, mCD14, CD16, CD86, and HLA-DR on sputum monocytes recovered from normal volunteers (Alexis et al. 2004a). We also reported an increase in the numbers of sputum monocytes (in addition to neutrophils), suggesting that O_3_ exposure resulted in an influx of activated monocytes. These data are supported by a recent animal study showing that O_3_ enhanced the expression of interstitial lung cell-surface molecules associated with antigen presentation and increased the number of antigen-presenting cells in the lung (Koike and Kobayashi 2004). In the present study, we found that 2 mg inhaled FP decreased the expression of CD11b, mCD14, CD16, CD64, CD86, and HLA-DR on sputum monocytic cells after O_3_ challenge compared with placebo treatment. The 0.5 mg dose of FP decreased the expression of CD86 and HLA-DR on sputum monocytes after O_3_ challenge compared with placebo. Given that these surface molecules are involved with mediating innate immune responses (CD11b and mCD14), acquired immune responses (CD16, CD64), and antigen presentation (CD86, HLA-DR), we speculate that O_3_ exposure may play a role in modifying how airway cells respond to a number of pathologic agents in the airborne environment. It is unclear whether the effect of FP on O_3_-induced changes in monocytic cell populations is due to effects on monocytes present in the airway when exposure began, or on the subsequent influx of monocytes that are activated from the circulation. Decreased monocytic cell influx could be mediated by an effect of FP on production of monocyte-associated chemotactic factors or decreased adhesion molecule expression on endothelial cells lining the postcapillary venules.

The use of CCP16 as a systemic marker for injury of the epithelium has been examined by several investigators (Blomberg et al. 2003; Helleday et al. 2006). O_3_ exposure is associated with increased serum levels of CCP16 (Blomberg et al. 2003). We likewise observed that O_3_ exposure caused an increase in serum CCP16 and further showed that pretreatment with either 0.5 mg or 2 mg inhaled FP prevented the O_3_-induced increase in CCP16. We compared CCP16 with SP-D, an innate immune molecule produced by type II alveolar epithelial cells and Clara cells. We previously showed that SP-D plays a protective role in O_3_-induced injury of the lung (Kierstein et al. 2006), but whether release of this protein into the circulation could parallel the inflammatory airway changes was unclear. Our study showed that CCP16 is a superior serum marker for injury of the airway epithelium and is a more sensitive biomarker for the effect of inhaled FP on airway inflammation compared with SP-D or, indeed, compared with the wide range of cytokines, chemokines, and inflammatory mediators we investigated.

The apparent discrepancy we observed between serum SP-D and CCP16 was likely influenced by many factors, including changes in lung concentrations. We previously showed that intracellular SP-D mRNA and protein expression are very sensitive to corticosteroids, cAMP, and cytokine levels (Cao et al. 2004) and that SP-D in broncho-alveolar lavage fluid is subject to rapid break-down after O_3_ exposure of mice (Kierstein et al. 2006). Although no formal comparisons have been made between local (pulmonary) expression of SP-D and CCP16, we speculate that the CCP16 molecule is more resistant to O_3_-induced breakdown than is SP-D. This is supported by the fact that CCP16 serum levels in a number of animal and human studies accurately reflected the extent of increases in capillary permeability after acute exposure to lipopolysaccharide, chlorine, or O_3_ (Lakind et al. 2007; Michel et al. 2005). Thus, the discrepancy we observed between serum levels of SP-D and CCP16 after O_3_ exposure may be due to different structure, regulation of expression, and sensitivity to O_3_-induced molecular changes. This discrepancy highlights the specific importance of CCP16 as a biomarker for lung injury and treatment effectiveness.

Overall, our observations are consistent with the hypothesis that FP will inhibit acute airway inflammation due to O_3_ exposure in a dose-dependent fashion that includes the therapeutic dose of 0.5 mg FP. Apart from percent PMNs, however, several of our find-ings with 0.5 mg FP did not reach statistical significance. This was likely due to an insufficient number of subjects examined at this dose. Subsequent studies using the 0.5 mg dose of FP will require a larger sample size. It is also possible that a more prolonged pre-treatment with FP before O_3_ challenge would have resulted in a more pronounced effect of 0.5 mg FP on airway inflammation, but our single-use administration of FP provided adequate drug exposure over the challenge time. We also note that this study was conducted in normal healthy volunteers. We chose healthy volunteers to avoid the potentially high variation in baseline inflammation associated with subjects with preexisting airway disease. Lower variability in healthy subjects would reduce the need to examine a large cohort of subjects in this study and allow us to attain an initial proof of pharmacology for new anti-inflammatory chemical entities. One cannot rule out, however, that because asthmatics have been reported to have an increased pulmonary sensitivity to O_3_ exposure (Alexis et al. 2000b; Scannell et al. 1996), although we did not observe a statistically significant effect using a single administration of 0.5 mg FP on airway inflammation, this might have been observed in a cohort of asthmatics. As noted above, Vagaggini et al. (2001) reported a significant inhibition of O_3_-induced inflammation in asthmatics with treatment of 400 μg budesonide administered twice daily. Using endotoxin as an inflammatory stimulus, we previously observed that 440 μg FP for 2 weeks delivered via a metered dose inhaler twice daily inhibited the effect of endotoxin on neutrophilic airway inflammation in allergic asthmatics (Alexis and Peden 2001). Thus, implementation of a longer treatment period in healthy individuals may have resulted in demonstration of anti-inflammatory efficacy of a single 500 μg dose of inhaled FP.

## Conclusion

We confirmed original observations that O_3_-induced airway neutrophilic inflammation was inhibited by a single administration of 2 mg FP and extended the findings by demonstrating decreased neutrophilic inflammation with the 0.5 mg FP treatment, as well. We also observed that both doses of FP inhibited the up-regulatory effect of O_3_ on airway monocytic cell-surface phenotypes and that 2 mg FP inhibited serum levels of CCP16. Taken together, these observations suggest that brief treatments with inhaled corticosteroids by persons in anticipation of exposure to air pollution may offer protection against the inflammatory effects of ambient air O_3_, particularly for those individuals with preexisting airway disease. However, it is important to note that inhaled corticosteroids had no protective effect on the spirometric decrements induced by O_3_, suggesting this component of airway function, particularly in individuals with preexisting airway disease, remains susceptible to the modifying effects of O_3_ exposure. A second conclusion is that O_3_ challenge with subsequent analysis of airway sputum and serum CCP16 is a good acute disease model for phase I screening of novel anti-inflammatory agents intended for use in asthma and COPD.

## Correction

In the original manuscript published online, Brad Harris was not included as an author. His name has been added here.

## Figures and Tables

**Table 1:** Subject demographics (*n* = 17).

Characteristic	Mean ± SE
Age (years)	26.4 ± 1.8
Sex	
Female	9
Male	8
Race	
Caucasian	10
African American	3
American Hispanic	2
Asian	1
Other	1
Height (cm)	170 ± 2.6
Weight (kg)	78 ± 3.9

**Table 2:** Mean (± SE) pulmonary function, eNO, and IOS.

				IOS		
	FEV_1_ (L)	FVC (L)	eNO (ppb)	R5	X5	*F*_res_ (Hz)
Baseline						
Pretreatment	3.76 ± 0.18	4.69 ± 0.21	12.54 ± 1.55	0.376 ± 0.008	−0.112 ± 0.003	12.27 ± 0.30
Placebo						
Preexposure (0 hr)	3.84 ± 0.01	4.70 ± 0.02	11.49 ± 1.26	0.409 ± 0.007	−0.161 ± 0.002	12.03 ± 0.28
Immediately after exposure	3.52 ± 0.07	4.39 ± 0.08	13.51 ± 1.39			
1 hr postexposure	3.71 ± 0.05	4.60 ± 0.06	14.24 ± 1.49	0.379 ± 0.008	−0.099 ± 0.002	11.85 ± 0.27
2 hr postexposure			13.76 ± 1.43	0.404 ± 0.04	−0.102 ± 0.01	11.87 ± 1.14
3 hr postexposure			8.94 ± 1.15	0.414 ± 0.04	−0.221 ± 0.11	12.60 ± 1.38
0.5 mg FP						
Preexposure (0 hr)	3.84 ± 0.02	4.75 ± 0.03	11.65 ± 1.91	0.383 ± 0.007	−0.112 ± 0.002	12.10 ± 0.24
Immediately after exposure	3.51 ± 0.05	4.43 ± 0.05	14.80 ± 1.68			
1 hr postexposure	3.69 ± 0.04	4.60 ± 0.05	15.53 ± 1.70	0.356 ± 0.007	−0.106 ± 0.002	12.01 ± 0.25
2 hr postexposure			15.85 ± 1.60	0.382 ± 0.05	−0.101 ± 0.01	12.26 ± 1.43
3 hr postexposure			12.16 ± 1.2	0.393 ± 0.05	−0.196 ± 0.08	12.99 ± 1.76
2.0 mg FP						
Preexposure (0 hr)	3.73 ± 0.02	4.61 ± 0.02	10.81 ± 1.75	0.382 ± 0.008	−0.113 ± 0.003	12.23 ± 0.25
Immediately after exposure	3.51 ± 0.07	4.35 ± 0.08	13.84 ± 1.32			
1 hr postexposure	3.60 ± 0.04	4.41 ± 0.05	14.87 ± 1.57	0.367 ± 0.007	−0.109 ± 0.003	11.70 ± 0.24
2 hr postexposure			13.65 ± 1.18	0.365 ± 0.04	−0.099 ± 0.01	11.47 ± 1.07
3 hr postexposure			11.88 ± 1.45	0.373 ± 0.04	−0.188 ± 0.08	11.45 ± 1.07

Abbreviations: R5, total respiratory resistance (cm H_2_O/L/sec); X5, reactance (cm H_2_O/L/sec).
